# Whole exome and transcriptome analysis revealed the activation of ERK and Akt signaling pathway in canine histiocytic sarcoma

**DOI:** 10.1038/s41598-023-35813-1

**Published:** 2023-05-25

**Authors:** Hajime Asada, Akiyoshi Tani, Hiroki Sakuma, Miyuki Hirabayashi, Yuki Matsumoto, Kei Watanabe, Masaya Tsuboi, Shino Yoshida, Kei Harada, Takao Uchikai, Yuko Goto-Koshino, James K. Chambers, Genki Ishihara, Tetsuya Kobayashi, Mitsuhiro Irie, Kazuyuki Uchida, Koichi Ohno, Makoto Bonkobara, Hajime Tsujimoto, Hirotaka Tomiyasu

**Affiliations:** 1 Department of Veterinary Internal Medicine, the University of Tokyo, Bunkyo-ku, Tokyo, Japan; 2 Department of Veterinary Pathology, the University of Tokyo, Bunkyo-ku, Tokyo, Japan; 3 Anicom Specialty Medical Institute Inc., Shinjuku-ku, Tokyo, Japan; 4 Veterinary Medical Center, the University of Tokyo, Bunkyo-ku, Tokyo, Japan; 5 Japan Small Animal Cancer Center, Tokorozawa, Saitama Japan; 6 Shikoku Veterinary Medical Center, Kita-gun, Kagawa Japan; 7 Department of Veterinary Clinical Pathology, Nippon Veterinary and Life Science University, Musashino, Tokyo Japan; 8 Present Address: Department of Obstetrics and Gynecology, The University of Chicago, Chicago, IL 60637 USA

**Keywords:** Haematological cancer, Bioinformatics

## Abstract

Histiocytic sarcoma (HS) is an incurable aggressive tumor, and no consensus has been made on the treatment due to its rare occurrence. Since dogs spontaneously develop the disease and several cell lines are available, they have been advocated as translational animal models. In the present study, therefore, we explored gene mutations and aberrant molecular pathways in canine HS by next generation sequencing to identify molecular targets for treatment. Whole exome sequencing and RNA-sequencing revealed gene mutations related to receptor tyrosine kinase pathways and activation of ERK1/2, PI3K-AKT, and STAT3 pathways. Analysis by quantitative PCR and immunohistochemistry revealed that fibroblast growth factor receptor 1 (FGFR1) is over-expressed. Moreover, activation of ERK and Akt signaling were confirmed in all HS cell lines, and FGFR1 inhibitors showed dose-dependent growth inhibitory effects in two of the twelve canine HS cell lines. The findings obtained in the present study indicated that ERK and Akt signaling were activated in canine HS and drugs targeting FGFR1 might be effective in part of the cases. The present study provides translational evidence that leads to establishment of novel therapeutic strategies targeting ERK and Akt signaling in HS patients.

## Introduction

Histiocytic sarcoma (HS) is the most aggressive subtype of histiocytic neoplastic disorders in humans originating from histiocytic cell lineages^1^. This is an uncommon malignancy accounting for < 1% of all hematopoietic neoplasms that affects a variety of organs including soft tissues, gastrointestinal tract, spleen, and lymph nodes with a high rate of distant metastasis^2^. In terms of genetic findings, activating mutations in genes related to the RAS-ERK and PI3K-AKT signaling pathways were identified by a targeted exome sequencing^3^, and activating mutations in RAS/RAF/MAPK pathway were reported in all of the 21 HS patients by whole exome sequencing (WES) and RNA sequencing (RNA-seq)^4^. Due to the rare occurrence, no consensus has been made on the treatment for the disease in human medicine leading to median survival time 52 months^5^. Therefore, there has been a pressing need to better understand the molecular basis in the disease and to develop more effective therapeutic strategies for improvement of outcome in patients.

Dogs have been advocated as potential translational models for studying human HS in recent years^6–8^, since this is the only well-recognized species that frequently develops sporadic HS and several cell lines have been established from clinical patients. Canine HS also affects a variety of organs^9^ with a high rate of systemic metastasis of 70–90%^10,11^ and is characterized by an aggressive biological and clinical behavior with a median overall survival time < 100 days^9,12^. Similar to human counterpart, mutations in *PTPN11* encoding *SHP2*, a tyrosine phosphatase required for MAPK pathway, were observed in 37% cases in Bernese Mountain dogs, one of the most predisposed breeds^8^. Also, we previously reported mutations in *TP53* in 46% HS cases affected by HS^13^. However, the genetic landscape of aberrant pathways is unclear in canine HS. Comprehensive analyses of aberrant molecular pathways are expected to establish the molecular basis in canine HS and, as a spontaneous model, to provide translational evidence of novel therapeutic targets in human counterpart.

Here, the present study aimed for revealing aberrated molecular pathways in canine HS by WES and RNA-seq analyses, followed by quantitative PCR (qPCR), immunohistochemistry (IHC), and several in vitro assays for further elucidation. The achievements of this study provide translational insights into development of novel therapeutic strategies in HS.

## Results

### Canine HS harbors mutations in receptor tyrosine kinase (RTK) signaling pathways-related genes

To comprehensively investigate genetic aberrations in canine HS, we first performed WES using genomic DNA extracted from five tumor and matching normal cell samples. Read count generated by WES, mapping rate, and coverage are shown in Additional file 1: Table S1. As a result, 35 mutations were identified and validated by Sanger sequencing in five dogs including *TP53*, *PDGFRB*, *PTPN11*, and *SH3KBP1* (Table 1). These mutations were confirmed to be somatic ones, because they were not observed in normal cell sample of each case by Sanger sequencing. To explore biological impacts and pathways where mutated genes were enriched, the mutated gene list was subjected to an online database for enrichment analysis. Consequently, PDGFR signaling pathway, positive regulation of ERK1/2 cascade, and positive regulation of PI3K signaling were included as significantly enriched GO terms (Table 2). In KEGG pathway analysis, PI3K-AKT signaling and RAS signaling pathways were called. These results together suggested that canine HS cells have mutations in genes associated with RTK, ERK1/2, and PI3K-AKT signaling pathways.

**Table 1:** Gene mutations identified in whole exome sequencing and validated by Sanger sequencing.

Dog	Gene name	Gene ID	Amino acid change	Chromosome
Dog 1	*CCDC136*	ENSCAFG00000023734	p.R870W	14
	*TP53*	ENSCAFG00000016714	p.I151fs	5
Dog 2	*BBX*	ENSCAFG00000009867	p.D53Y	33
	*N4BP2*	ENSCAFG00000015929	p.S1282F	3
	*PDGFRB*	ENSCAFG00000018214	p.D881_I882delinsV	4
Dog 3	*ATRX*	ENSCAFG00000017252	p.L2119fs	X
	*GARNL3*	ENSCAFG00000020142	p.R204*	9
	*HTR2C*	ENSCAFG00000029120	p.R156H	X
	*NR3C2*	ENSCAFG00000007813	p.R780*	15
	*NRXN3*	ENSCAFG00000030472	p.V262A	8
	*SH3KBP1*	ENSCAFG00000013075	p.R423*	X
	*TP53*	ENSCAFG00000016714	p.D11fs	5
Dog 4	*NAV3*	ENSCAFG00000005746	p.A343T	15
	*PTPN11*	ENSCAFG00000008894	p.G503V	26
	*ZMYM3*	ENSCAFG00000017043	p.R295C	X
Dog 5	*AKAP4*	ENSCAFG00000015984	p.Q669*	X
	*ASMT*	ENSCAFG00000024670	p.G168R	X
	*C7*	ENSCAFG00000018608	p.L726S	4
	*CLCA4*	ENSCAFG00000020244	p.M325T	6
	*COL11A1*	ENSCAFG00000019985	p.P1193T	6
	*FBN1*	ENSCAFG00000014548	p.E2759G	30
	*FRAS1*	ENSCAFG00000008752	p.I1313fs	32
	*FREM2*	ENSCAFG00000006019	p.D1252Y	25
	*GGNBP2*	ENSCAFG00000017839	NA	9
	*LAMA1*	ENSCAFG00000018597	p.S3027F	7
	*MLEC*	ENSCAFG00000010408	p.E109K	26
	*MTX2*	ENSCAFG00000013416	p.L157_Y159del	36
	*NUGGC*	ENSCAFG00000008347	p.R651fs	25
	*OPCML*	ENSCAFG00000009842	p.N40H	5
	*PDGFD*	ENSCAFG00000032342	p.S265T	5
	*PTPN11*	ENSCAFG00000008894	p.E76K	26
	*RELN*	ENSCAFG00000025345	p.I819M	18
	*RIPK1*	ENSCAFG00000009321	p.L659fs	35
	*SLC6A13*	ENSCAFG00000015769	p.R413H	27
	*SMARCAL1*	ENSCAFG00000014421	p.R652C	37

**Table 2:** Results of enrichment analysis in whole exome sequencing.

	*P* value
GO terms	
Platelet-derived growth factor receptor signaling pathway	6.3E−04
Positive regulation of ERK1 and ERK2 cascade	1.2E−03
Multicellular organism development	1.3E−02
Morphogenesis of an epithelium	1.9E−02
Peptidyl-tyrosine phosphorylation	2.7E−02
Replication fork processing	2.7E−02
Cell communication	3.4E−02
Inner ear development	3.4E−02
Multicellular organism development	4.0E−02
Positive regulation of phosphatidylinositol 3-kinase signaling	7.7E−02
KEGG pathway	
PI3K-Akt signaling pathway	2.8E−04
Focal adhesion	4.9E−04
Melanoma	7.9E−03
ECM-receptor interaction	1.2E−02
Prostate cancer	1.2E−02
Gap junction	1.2E−02
Ras signaling pathway	6.4E−02

### RTK signaling pathways are activated in canine HS

**Figure 1:**
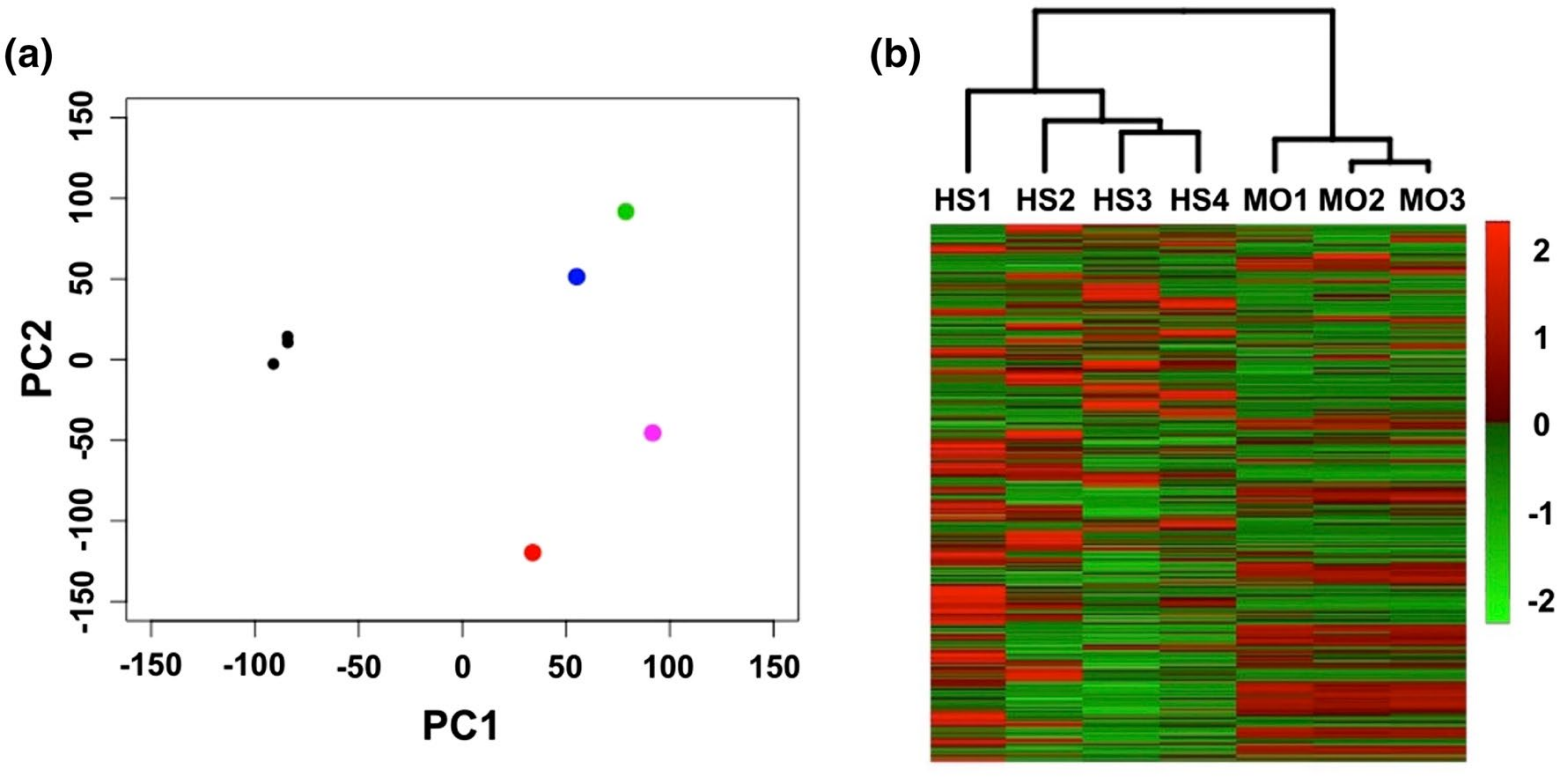
Comparisons of gene expression profiles between canine histiocytic sarcoma (HS) cell samples and peripheral blood monocytes (PBMoC) of a healthy beagle. (**a**) Results of principal component analysis. The colored points represent each HS cell samples and black points represent each PBMoC technical replicates of a healthy beagle. (**b**) Results of hierarchical clustering. These results yielded two distinct clusters composed of HS samples and PBMoC technical replicates of a healthy beagle, respectively.

To assess activation or inactivation profiles of signaling pathways in HS cells, we next performed RNA-seq using four tumor samples. Its performance metrics are shown in Additional file 1: Table S1. First, gene expression profiles (GEPs) were compared by principal component analysis and hierarchical clustering. These yielded two distinct clusters composed of HS patients and peripheral blood CD14^+^ monocytes (PBMoC), which was prepared as technical replicates, respectively (Fig. 1). When compared GEPs between HS and PBMoC, 1472 DEGs were extracted, and the expressions of 1231 genes were significantly increased and those of 241 genes were significantly decreased in HS tissues [see Supplementary dataset file 1 and Supplementary dataset file 2]. Next, enrichment and upstream regulator analyses were performed using expression profiles of these DEGs. In the enrichment analysis, 83 GO terms were called as significant [see Supplementary data set file 3], and these terms included positive regulation of MAPK activity (Table 3). In KEGG pathway analysis, 27 pathways were significantly enriched [see Supplementary data set file 3], and these pathways included pathways in cancer, PI3K-AKT signaling pathway, and transcriptional misregulation in cancer (Table 3). To identify key molecules that regulate aberrant pathways, upstream regulator analysis was then conducted. Consequently, 94 and 40 genes were respectively identified as activated or inhibited upstream regulators [see Supplementary data set file 4]. The activated upstream regulators included those related to RTK signaling (*EGFR*, *ERBB2*, *FGFR1*, *RAF1*, *ERK1/2*, *PI3K*, *AKT*, *STAT3, VEGF*, *VEGFA*, *EGF*, and *FGF2*). *FOXM1*, a transcription factor whose expression is induced via overexpression of RTKs^14^, was also called as activated. The 40 inhibited regulators included *CDKN1A* and *FAS*, genes related to cell cycle and apoptosis. WES and RNA-seq together suggested that RTK-ERK/AKT/STAT3 signaling pathways were activated in HS cells via mutations in their regulator genes, and RTKs such as EGFR, ERBB2, and FGFR1 were suggested to be upstream regulators.

**Table 3:** Representative results of enrichment analysis in RNA-seq.

	*P* value
GO term	
Positive regulation of MAP kinase activity	2.2E−02
KEGG pathway	
Pathways in cancer	2.1E−07
PI3K-Akt signaling pathway	7.3E−05
Transcriptional misregulation in cancer	2.4E−03
MicroRNAs in cancer	5.3E−03
Basal cell carcinoma	1.1E−02
Small cell lung cancer	1.9E−02
Melanoma	4.7E−02

### FGFR1 is highly expressed in canine HS

**Figure 2:**
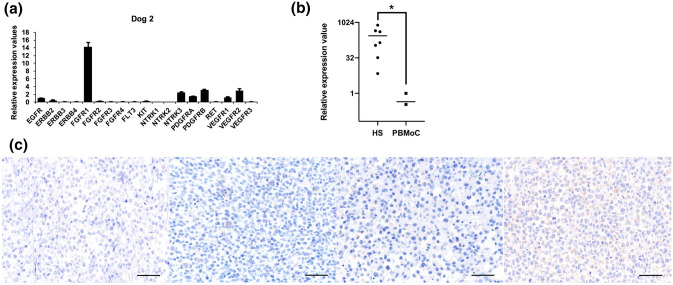
Expression of FGFR1 in canine histiocytic sarcoma (HS) cases. (**a**) Comparison of mRNA expressions among receptor tyrosine kinase (RTK) genes in Dog 2. *FGFR1* mRNA expression was the highest among RTK genes. *RPL32* was used as an internal control gene. Each experiment was conducted in triplicate. (**b**) Comparisons of *FGFR1* mRNA expressions between canine HS cases and peripheral blood monocytes (PBMoC) of healthy beagles using Mann–Whitney *U* test. *FGFR1* mRNA expressions were higher in canine HS cases than those in PBMoCs. The expression level was below detection limit in one of the three healthy dogs. *RPL32* was used as an internal control gene. Each experiment was conducted in triplicate. *P = 0.017. (**c**) Expression of FGFR1 protein in canine HS tissue examined by immunohistochemistry. The representative results of negative control, weak, moderate, and strong expressions are shown in order from left to right. Scale bars = 50 µm.

To identify target upstream molecules for effective treatment of canine HS, mRNA expression levels of RTKs were assessed. First, normalized expression values for 19 RTKs were extracted from RNA-seq data. As a result, there was a tendency that expression of *FGFR1* and *PDGFRB* were found to be higher than other RTK genes [see Additional file 1: Table S2]. To elucidate high expression of these RTKs, RT-qPCR was performed using RNA samples from seven dogs including those subjected to RNA-seq analysis. In this analysis, *RPL32* was used as an internal control gene. Consequently, expression level of *FGFR1* was the highest among RTKs in 5 of the 7 samples (Fig. 2a and Additional file 1: Fig. S1). In comparison of *FGFR1* expression between HS and PBMoC, its expression was significantly higher in HS (Fig. 2b). These data suggested that *FGFR1* gene was over-expressed in canine HS and functioned as an upstream regulator of ERK1/2 and PI3K-AKT pathways. To further elucidate high expression of FGFR1, we performed IHC using formalin-fixed paraffin-embedded (FFPE) tissues from 13 dogs including 9 not subjected to other analyses above [see Additional file 1: Table S3]. Consequently, the expression of FGFR1 was observed in all samples. FGFR1 was strongly positive in 11/13 samples, moderately and weekly positive in 1 sample each (Fig. 2c).

### FGFR1 mRNA is highly expressed and ERK and Akt pathways were activated in canine HS cell lines

**Figure 3:**
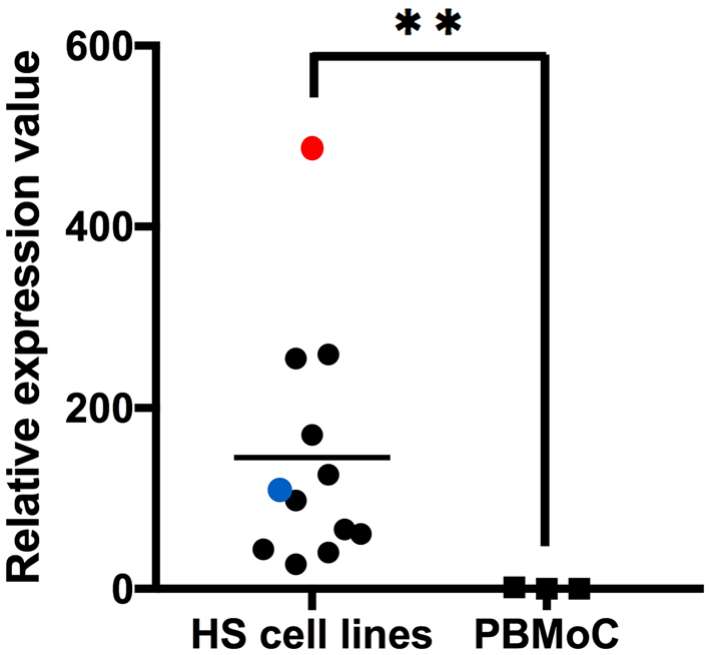
Comparisons of *FGFR1* mRNA expressions between canine histiocytic sarcoma (HS) cell lines and peripheral blood monocytes (PBMoC) of three independent healthy beagles as biological replicates using Mann–Whitney *U* test. *FGFR1* mRNA expressions were higher in canine HS cell lines than those in PBMoCs. DHS1 and DHS2 were shown in red and blue, respectively. **P = 0.0044.

**Figure 4:**
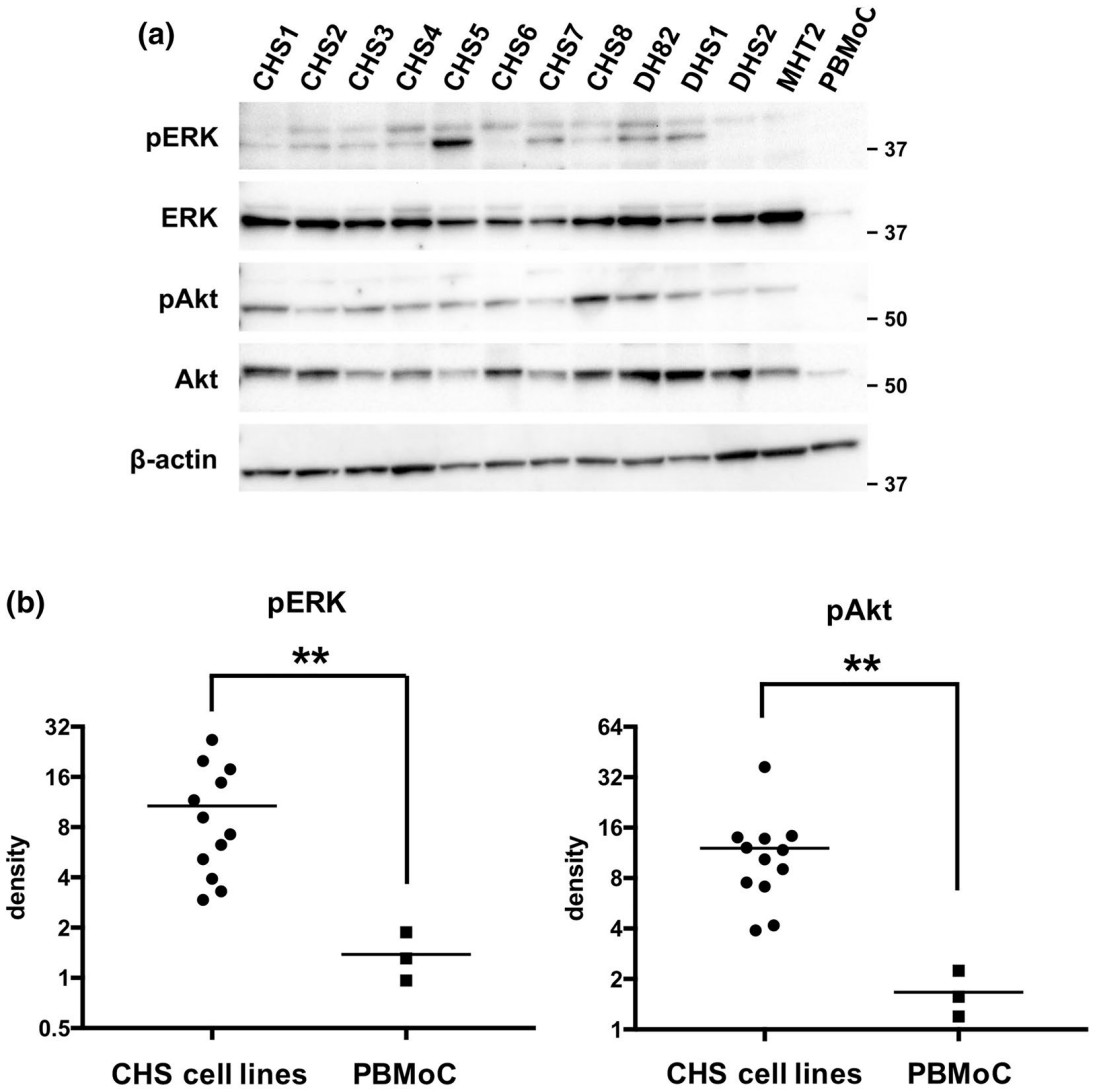
Comparisons of the amounts of phosphorylated ERK1/2 (pERK), ERK, phosphorylated Akt (pAkt), and Akt among canine histiocytic sarcoma (CHS) cell lines and peripheral blood monocytes (PBMoC). (**a**) The representative result of Western blot. This figure was prepared by cropping the images of the bands indicating each antigen from the figures shown in Additional file 1: Fig. S5. (**b**) The result of statistical comparison by density analysis for Western blot in triplicate. The amounts of pERK and pAkt were significantly higher in CHS cell lines than PBMoC. **P = 0.0044.

Motivated by above data, targeting FGFR1 was hypothesized to be an effective strategy for treatment of canine HS. First, RT-qPCR were performed to evaluate *FGFR1* expression in 12 cell lines, and its expressions in these cell lines were compared with PBMoC from three healthy beagles. As a result, *FGFR1* mRNA was significantly highly expressed in canine HS cell lines compared with PBMoC (Fig. 3). Next, we examined intracellular activation of ERK and Akt signaling using 12 canine HS cell lines and PBMoC by Western blot, and the results showed that the amounts of phosphorylated ERK and Akt were significantly increased in canine HS cell lines compared to PBMoC (Fig. 4). We could not examine FGFR1 protein expressions by Western blot in the cell lines, because we could not verify the specificities against canine FGFR1 in all antibodies we tried: we observed many non-specific bands and could not see the band corresponding to FGFR1 and we could not find any suitable positive control, cell line expressing canine FGFR1.

### FGFR1 inhibitor inhibits cell-growth and induce apoptosis in part of canine HS cell lines

**Figure 5:**
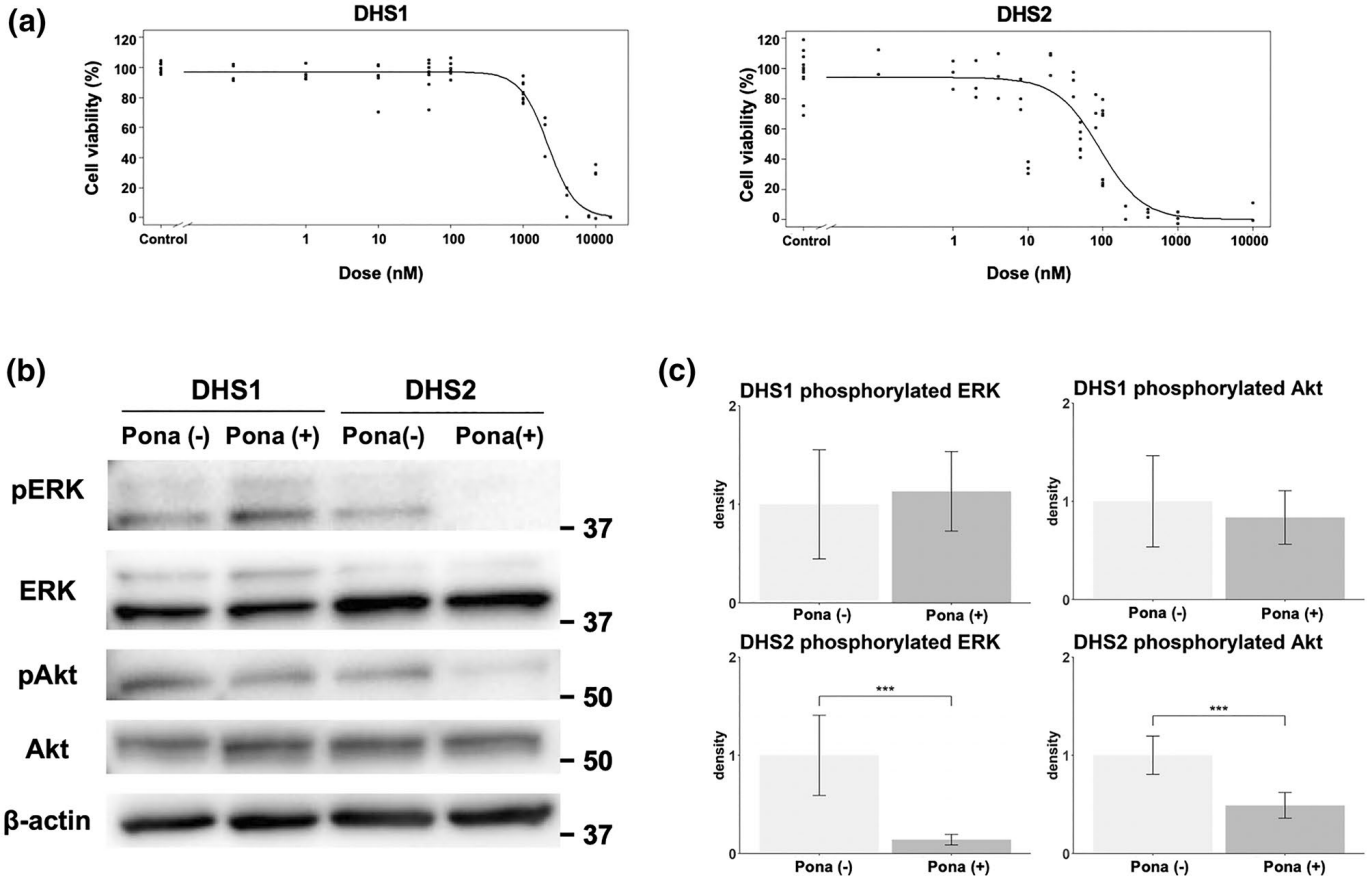
Effects of tyrosine kinase inhibitors that targets FGFR1, ponatinib. (**a**) Growth inhibitions by ponatinib in canine histiocytic sarcoma cell lines, DHS-1 and DHS-2. (**b**) The representative figure that shows changes in the amounts of phosphorylated ERK1/2 (pERK) and AKT (pAkt) after treatments of ponatinib (Pona). This figure was prepared by cropping the images of the bands indicating each antigen from the figures shown in Additional file 1: Fig. S6. (**c**) The result of statistical comparison by density analysis for Western blot in triplicate. Although the amounts of pERK and pAKT were not changed after treatment with Pona in DHS1, those in DHS2 were decreased by treatment with ponatinib. ***P < 0.0001.

Finally, we evaluated the efficacy of FGFR1 inhibitor in vitro using cell lines derived from canine HS. In cell viability assays using tyrosine kinase inhibitors that target FGFR1, ponatinib, the inhibitor showed dose-dependent growth inhibitory effects in all 12 HS cell lines but did not show such effect in a non-HS cell line (MDCK) (Fig. 5a and Additional file 1: Fig. S2), implying that FGFR1 inhibition impairs viability of canine histiocytic cells. DHS-2 was most sensitive to ponatinib and IC_50_ value was 90.4 nM in this cell line [see Additional file 1: Table S4] which was within the clinically relevant plasma concentration in human patients (under than 145 nM)^15^. CHS-6 was secondary sensitive to ponatinib and IC_50_ value was also within the concentration. Meanwhile, IC_50_ value in other HS cell lines were above the clinically relevant plasma concentration.

When examined the induction of apoptosis by ponatinib treatment, the proportion of apoptotic cells was increased in only one of the twelve cell lines [see Additional file 1: Fig. S3], showing that apoptosis may not be the main mechanism of cell killing from ponatinib. The cell lines were serum starved, and then treated with ponatinib. Although the amounts of phosphorylated ERK1/2 and Akt were not changed after treatment of 1 μM ponatinib in DHS1, phosphorylation of ERK1/2 and Akt were significantly inhibited in DHS2, where the IC_50_ values were lower than DHS1, by treatment of 1 μM ponatinib (Fig. 5b,c).

## Discussion

HS is an uncommon aggressive tumor in humans. Although some mutations related to RAS-ERK and PI3K-AKT pathways have been described^3,16–18^, no consensus has been made on the molecular-targeted treatment for the disease to date. This was considered due to the lack of detailed analysis of genetic aberrations based on genome-wide analyses. To comprehensively explore somatic gene mutations in the disease, therefore, we first performed WES in naturally occurring canine HS, an advocated translational model. As a result, mutations were identified in genes related to RTK pathways including *PDGFRB* (Dog 2), *PTPN11* (Dog 4 and 5), and *SH3KBP1* (Dog 3). PDGFRB is a receptor of platelet derived growth factor and induce RAS-MAPK, PI3K-AKT pathways upon ligand stimulation to promote cellular proliferation and survival. Mutations in the gene have been identified in rare neoplasms in human such as infantile myofibroma^19^ and intimal sarcoma^20^. In addition, rearrangement of *PDGFRB* leading to production of fusion proteins has been identified in myeloid and lymphoid neoplasms^21^. Mutations in *PTPN11* were also frequently identified in the disease in a specific canine breed^8^. The gene encodes SHP2, a tyrosine phosphatase that regulate phosphorylation of RAS, and canine HS cell lines harboring *PTPN11* mutations were sensitive to SHP2 inhibitors^22^. Dog 3 harbored a mutation in *SH3KBP1* which is also named CIN85. CIN85 is an adaptor protein involved in the downregulation of RTKs^23^. Mutations in the gene might lead to over-expression of RTKs in HS cells. In the following enrichment analysis, mutations were significantly enriched in PDGFRB, ERK1/2, and PI3K-AKT signaling pathways. Consistent with previous reports on gene mutations in human or canine HS^3,8,13,16–18^, these results indicate that HS harbors mutations in genes relating to RTK-ERK/AKT signaling pathways. RTK signaling pathways were reported to enable sustained cell proliferation, which is one of hallmarks of cancer^24,25^, and this characteristics is not specific to HS. We also revealed the mutations in the genes that are not related to RTK signaling pathways but are thought to be cancer hallmarks. Dog 3 and dog 5 harbored mutations of *ATRX* and *SMARCAL1*, which were reported to be related to replicative immortality^25,26^. Dog1 and Dog 3 harbored mutations of TP53, which is important component relating to resisting cell death^25^. The mutations in these genes might be also involved in the development of HS. Future investigation is needed to clarify the frequency of these mutations in a larger number of canine HS patients.

For establishment of effective molecular targeted therapies, aberrant signaling pathways need to be explored as well as gene mutations. Therefore, we next performed RNA-seq and compared the data from HS specimens with those from PBMoC. Consequently, in the enrichment analysis, DEGs were significantly enriched in MAPK activity and PI3K-AKT signaling pathways. Upstream regulator analysis also identified several RTK signaling pathways-related molecules as activated regulators such as EGFR, ERBB2, FGFR1, RAF1, ERK1/2, PI3K, AKT, and STAT3. This indicates that RTK-ERK/AKT/STAT3 signaling pathways are activated in HS cells. This is supported by the predicted activation of FOXM1, a transcription regulator of cell cycle induced via activated ERK/AKT pathways^14^. FOXM1 has been reported to be activated in various cancers and its knockdown or inhibition showed therapeutic effects on various cancers^27,28^, suggesting that FOXM1 might be an effector molecule that plays roles in neoplastic phenotypes in HS cells. Further investigations are needed to clarify its association with the activation of RTK pathways and the efficacy of its inhibition in the disease. Activation of RTK pathways is also supported by previous reports on clinical or preclinical efficacies of BRAF and MEK inhibitors in human and canine HS^6,16^. On the other hand, K-RAS, H-RAS, and N-RAS were called as inhibited upstream regulators. These are small GTPases activated by ligand-stimulated RTKs and trigger downstream ERK1/2 and PI3K-AKT pathways, and the previous studies identified activating mutations in RAS in human HS^17,18^. In human medicine, acquired resistance to RTK inhibitors were reported during treatment regimen because of mutations in RAS^29,30^. In a part of those cases, oncogenic RAS inhibitors might be alternative therapeutic strategies. Although no RAS mutation was found in the present study, the evaluation of gene mutations including RAS should be important also in canine HS during treatments using RTK inhibitors. In the present study, it is possible that K-RAS, H-RAS, and N-RAS were statistically called as inhibited due to inactivation of other various downstream pathways than ERK/AKT signaling that were not related to neoplastic phenotypes. Another possibility is that RTKs trigger ERK/AKT pathways independently of RAS in canine HS cells. Further investigation is needed for better understanding of the interactions among these molecular pathways and their biological functions in HS cells.

Among upstream regulatory molecules identified by RNA-seq, RTKs including EGFR, ERBB2, and FGFR1 were considered significant target for treatment that trigger downstream ERK1/2, AKT, and STAT3 signaling pathways, and expression data from RNA-seq indicated high expression of *FGFR1* mRNA in HS cells. Thus, we next assessed mRNA and protein expressions of RTKs by RT-qPCR and IHC, respectively. In RT-qPCR, expression level of *FGFR1* mRNA was the highest among RTK genes in five of the seven canine HS specimens. In addition, its expression level was significantly higher in HS than PBMoC. Moreover, IHC demonstrated expression of FGFR1 in all 13 HS tissues examined. These findings implied that over-expression of FGFR1 plays roles in pathophysiology of the disease and is a promising target for treatment. In the present study, we used PBMoC of healthy dogs as controls to compare *FGFR1* mRNA expression levels with canine HS cells, because we could not obtain the matched normal tissue or peripheral blood of each case due to the nature of retrospective sample collection. As another limitation, three PBMoCs used for RNA-seq analysis were collected from a healthy control dog as technical replicates. Although RT-qPCR validations were conducted using three PBMoCs collected from three independent healthy control dogs, further confirmation of over-expression of FGFR1 should be performed using these matched normal samples in the future.

Based on data from integrated analysis, we hypothesized that FGFR1 signaling plays roles in viability of HS cells. To test the hypothesis, we evaluated the efficacy of a FGFR1 inhibitor in vitro using canine HS cell lines. A clinically applicable FGFR1 inhibitor, ponatinib, showed growth inhibitory effects in a dose dependent manner, accompanied by inducing apoptosis in one of the twelve CHS cell lines. These results indicated that FGFR1 signaling plays essential roles in cellular growth and survival of canine HS cells and its inhibition might be effective therapeutic strategy in some patients suffering from the disease. Clinical or preclinical efficacies of FGFR inhibitors have been described in several cancers including lung cancers^31,32^, malignant mesothelioma^33,34^, and acute myeloid leukemia^35,36^. In addition to direct effects on tumor cells, FGFR inhibition has been reported to target FGF signaling in the tumor microenvironment; it inactivates cancer-associated fibroblast^37^, prevents angiogenesis via inhibiting proliferation and migration of endothelial cells^38,39^, and inhibits tumor-infiltration of myeloid-derived suppressor cells^40–42^. Since hyperphosphatemia, diarrhea, fatigue, dermatologic, and ocular toxicities were frequently reported in human medicine^43^, preclinical study to evaluate adverse events should be conducted also in dogs.

It is the most important limitation in this study that we could not examine the FGFR protein expressions in canine HS cell lines due to the lack of suitable antibodies, and we could not confirm whether ponatinib exerted antitumor effects through the inhibitions of FGFR1. Furthermore, ponatinib was effective only in part of HS cell lines and the changes in the activation status of ERK and Akt pathways were various among cell lines. Therefore, further studies are needed to elucidate the molecular mechanisms associated with the differences in the effects of ponatinib and to investigate the effective treatment to inhibit the activations of ERK and Akt pathways in HS.

## Conclusions

In conclusion, our findings identified the activation of ERK and Akt signaling as a novel and potent target for treatment of canine HS through integrated whole exome and transcriptome investigations. Dogs frequently develop the disease compared with humans, and thus canine HS has been advocated as a translational animal model of naturally occurring HS. Therefore, the present study provides translational evidence that leads to establishment of novel therapeutic strategies targeting ERK and Akt signaling in HS patients.

## Methods

### Patient and healthy dog samples

Seventeen canine patients with HS were included in this study [see Additional file 1: Table S3]. These dogs were diagnosed by histological or cytologic evaluation. In histopathological examination, in addition to morphological and histopathological features as previously described^44^, reactivities to the antibodies against MHC class II, ionized calcium-binding adaptor molecule 1, or CD204 were examined by IHC for confirmation of histiocytic origin. The cytochemical staining for alpha-naphthyl butyrate esterase and inhibition of the enzyme by sodium fluoride were performed as markers of monocyte/macrophage lineage in a dog diagnosed based on a cytologic evaluation. At diagnosis, disseminated lesions were observed in twelve cases and localized lesions were found in five cases. Tumor cell samples were obtained from FFPE tissues (n = 9) or freshly frozen samples (n = 8). Matching normal cell samples were also obtained from normal tissues or peripheral blood (n = 5).

PBMoC were collected from healthy beagle dogs. Preparation of PBMoC was carried out with MACS cell separation system (Miltenyi Biotec, Germany) with an antibody against CD14 as previously reported in our laboratory^45^. Detailed information of antibodies used in this study is listed in Additional file 1: Table S5. For RNA-seq analysis, PBMoC was collected from a healthy beagle, and three PBMoC samples were collected at three different times as technical replicates. For qPCR analysis, PBMoC was collected from three healthy beagles, including the one used for RNA-seq analysis, as biological replicates. The median age and body weight of these healthy beagles were 7.4 (6.3–7.6) years old and 9.8 (9.5–11.5) kg, respectively, and all three dogs were castrated male.

### WES and data processing

Genomic DNA was extracted using DNeasy Blood and Tissue Kit (QIAGEN, The Netherlands) from the freshly frozen tumor and normal cell samples of five dogs. Exonic DNA fragments were captured using SureSelect Canine All Exon V2 (Agilent Technologies, USA), and whole exome sequencing (100-bp paired-end) was performed using NextSeq 500 (Illumina, USA) following the manufacturer’s instructions. Signal captures were converted into FASTQ files using bcl2fastq (v2.18.0.1) and then trimmed with Trimmomatic (v0.36). The research resource identifiers (RRIDs) for each software or data processing resources used in this study are listed in Additional file 1: Table S6. The alignment of processed reads to a canine reference genome (CanFam 3.1, GenBank assembly accession: GCA_000002285.2) was carried out using Bowtie2 (v2.2.9), and local realignment and variants calling in each sample were performed using the standard Genotype Analysis Toolkit (GATK). Somatic mutations were also called using VarScan2^46^ and annotated using SnpEff, and the called mutations were filtered by the read depth as previously reported^47^. Then, mutations identified by both GATK and VarScan2 were extracted to exclude false positive calling. Among these, single nucleotide variations were further subjected to Polyphen-2, SIFT, and PROVEAN for prediction of their functional impacts, and those predicted to be damaging by more than two algorithms were extracted and validated by Sanger sequencing. As for small insertions or deletions, those leading to frameshift and those predicted to be damaging by PROVEAN were extracted and validated. For Sanger sequencing, the extracted DNA samples were amplified by PCR with the primers listed in Additional file 1: Table S7. The products were directly sequenced using BigDye terminator v3.1/1.1 Cycle Sequencing Kit (Applied Biosystems, USA) and genetic analyzer (3130XL, Applied Biosystems). When the sequence could not be directly analyzed, PCR products were inserted into a T/A cloning vector (pGEM-T Easy, Promega, The Netherlands), and the vectors were transfected into competent cells (DH5α, TOYOBO, Japan). The plasmids extracted from the DH5α cells were subjected to sequence analysis as described above. At least 5 clones were sequenced to find mutated genes. When no mutation was found in the first 5 clones, additional 5 clones were sequenced. Finally, the list of validated mutations was subjected to enrichment analysis using DAVID Bioinformatics Resource (v6.8) to examine significant Gene Ontology (GO) term or enriched pathways.

### RNA-seq and data processing

Total RNA samples were extracted from tumor cell samples (n = 4) and PBMoC of a healthy beagle collected with three independent time using RNeasy Mini Kit (QIAGEN), and RNA integrity was examined using 2100 Bioanalyzer (Agilent Technologies) to confirm RNA integrity number > 8.

For RNA samples from tumor cell samples, total RNA (500 ng) was subjected to ribosomal RNA depletion using NEBNext rRNA Depletion Kit and poly-A purification using NEBNext Poly(A) mRNA Magnetic Isolation Module (New England BioLabs, USA). Then, sequencing libraries were prepared using NEBNext Ultra Directional RNA Library Prep Kit for Illumina (New England BioLabs), and RNA-seq (76-bp paired-end) was performed using NextSeq 500. As for RNA samples from PBMoC, total RNA (1 ng) was subjected to preparation of sequencing libraries using SMARTer Stranded RNA-seq Kit (Takara bio, Japan), which is a kit for RNA-seq using a low amount of RNA, and RNA-seq (150-bp paired-end) was performed using NovaSeq 6000 (Illumina).

Data processing was performed as previously described^48^. Differential gene expression analysis was performed using EdgeR to extract differentially expressed genes (DEGs). DEGs were defied as genes with log_2_ fold change > |1| and false discovery rate < 0.001. Then, normalized gene counts were imported into Java TreeView (v1.1.64) for hierarchical clustering analysis and visualization. Extracted DEGs were also subjected to enrichment and upstream regulator analyses using DAVID Bioinformatics Resource and Ingenuity Pathway Analysis (QIAGEN).

### RT-qPCR

Reverse transcription and real-time PCR were performed as described previously^49^. Primer sequences were designed using Primer3 (v0.4.0) or based on a previous report^50^ as listed in Additional file 1: Table S8. Amplifications were normalized to *RPL32*, determined as previously described^49,50^, and fold change was assessed by ΔΔC_T_ method. PCR efficiencies and R^2^ values for primer pairs were confirmed to be 90–110% and > 0.990, respectively.

### IHC

Thirteen FFPE HS tissue specimens were subjected to IHC of FGFR1. For each sample, 4 µm-thick sections were deparaffinized and rehydrated, and antigen retrieval was performed by autoclaving sections at 121 °C for 15 min in citrate buffer (pH 6.0). After cooling for 1 h, they were treated with 3% hydrogen peroxide-methanol at room temperature for 10 min to inactivate endogenous peroxidase. Then, they were incubated with 5% goat serum (Cedarlane Laboratories, Canada) in TBS-T at room temperature for 30 min to block nonspecific reactions. The sections were incubated with primary antibody against FGFR1 [see Additional file 1: Table S5] at 4 °C overnight and then with Envision horseradish peroxidase-labeled anti-rabbit IgG polymer (Agilent Technologies) at 37 °C for 30 min. The antibody used in this study was previously reported to react with canine FGFR1, whose amino acid sequence shows > 90% homology to human FGFR1^51^. The reacted products were visualized using Liquid DAB+ Substrate Chromogen System (Agilent Technologies), and counterstaining was conducted with Mayer’s hematoxylin (FUJIFILM, Japan). Negative control was prepared by omitting the primary antibody. Positive control was prepared using normal skin tissue of Dog 3 based on a previous study^52^ as shown in Additional file 1: Fig. S4. Each slide was histologically evaluated by two veterinary pathologists (M. Hirabayashi and JK. Chambers). The intensity of expression was scored in 3 grades: negative, weakly positive, and positive based on a previous report^53^.

### Cell culture and compounds

Twelve canine HS cell lines, DH82 (ATCC Cat# CRL-10390), DHS-1, DHS-2, MHT-2, CHS-1 to CHS-8, and MDCK were used in this study. DHS-1 and DHS-2 were originally established from Dog 1 and Dog 4, respectively, at the time of diagnosis, and expression of CD11c was confirmed by flow-cytometry prior to this study. CHS-1 to CHS-7^54^, MHT2^55^, CHS-8^22^, and MDCK^15^ were established in the previous studies. CHS-8, which was previously named as ROMA, was established and validated in the previous report^22^. All cell lines were regularly tested for mycoplasma contamination and were cultured at 37 °C in DMEM supplemented with 10% FBS (Thermo Fisher Scientific, USA) and 100 U/mL penicillin–streptomycin in a humidified atmosphere containing 5% CO_2_. Ponatinib was obtained from Selleck Chemicals, USA, and DMEM, penicillin–streptomycin, and DMSO were from FUJIFILM, Japan. Ponatinib was dissolved in DMSO and then diluted in fresh medium prior to use. Final concentration of DMSO in growth medium did not exceed 0.1% (v/v) when cells were treated, and equal amounts of DMSO were added to control conditions.

### Cell viability and apoptosis assays

The effect of pharmacologic FGFR1 inhibition on cell viability was assessed by a WST-8 method as previously reported^49^. Briefly, cells (2 × 10^4^ cells/mL) were seeded in 96-well microplates in triplicate, and then added with various concentrations of ponatinib. After 72 h, cell viability was measured using Cell Counting Kit-8 (Dojindo, Japan). Log-logistic curve and IC_50_ were obtained determined by dose–response analysis using R package drc (R 4.1.0, drc package 3.0.1). In an apoptosis assay, apoptotic cells were detected after incubation with 1 µM of ponatinib overnight using MEBCYTO Apoptosis Kit (Annexin V-FITC Kit) (Medical and Biological Laboratories, Japan) following the manufacturer’s instruction.

### Western blot

After serum starvation for 24 h using DMEM supplemented with 0% FBS for DHS-1 and 1% FBS for DHS-2, respectively, cells were treated with 1 µM of ponatinib for 3 h. Same amount of whole cell lysate samples extracted from these treated cells was separated by SDS-PAGE and blotted on PVDF membranes. After blocked with 5% bovine serum albumin (MilliporeSigma, USA), membranes were incubated with primary antibodies as shown in Additional file 1: Table S5. After incubation with primary antibodies, the membranes were washed and incubated with HRP-conjugated secondary antibody. Then, the membranes were incubated with Luminata Forte Western HRP Substrate (MilliporeSigma). Membranes were visualized using ChemiDoc XRS Plus (Bio-rad Laboratories, USA), and density of bands was analyzed by ImageJ software. Density analysis was conducted using the data obtained in triplicate.

### Statistical analysis

Computations were carried out using Prism (v.9.2.0) except for data processing of WES and RNA-seq as described above. Analytical methods used to assess statistical differences and *P* values are indicated in the figures and/or legends. Experimental data from primary samples were expressed as means ± SD of triplicate measurements, and those from cell lines were as means ± SD of three independent experiments. *P* value < 0.05 was considered significant in each statistical analysis unless otherwise specified. In an upstream regulator analysis using RNA-seq data, genes with z score < − 2.5 or > 2.5 were considered significant regulators and extracted.

### Ethics approval and consent to participate

All methods were carried out in accordance with relevant guidelines and regulations. All experimental protocols were approved by the animal care committee of the University of Tokyo (approval number, P16-172). All methods are reported in accordance with ARRIVE guidelines (https://arriveguidelines.org/) for the reporting of animal experiments. Written informed consent was obtained from all dog owners.

## Supplementary Information

Supplementary Information 1.

Supplementary Information 2.

Supplementary Information 3.

Supplementary Information 4.

Supplementary Information 5.

## Data Availability

All data generated or analyzed during this study except for those generated by WES and RNA-seq are included in this published article. Datasets obtained through WES and RNA-seq analyses are available at the DDBJ Sequenced Read Archive repository with accession number DRA010452 (https://ddbj.nig.ac.jp/resource/sra-submission/DRA010452), DRA013421 (https://ddbj.nig.ac.jp/resource/sra-submission/DRA013421) and DRA013450 (https://ddbj.nig.ac.jp/resource/sra-submission/DRA013450).

## References

[1] Swerdlow SH (2016). The 2016 revision of the World Health Organization classification of lymphoid neoplasms. Blood.

[2] Hornick JL, Jaffe ES, Fletcher CDM (2004). Extranodal histiocytic sarcoma: Clinicopathologic analysis of 14 cases of a rare epithelioid malignancy. Am. J. Surg. Pathol..

[3] Shanmugam V (2019). Identification of diverse activating mutations of the RAS-MAPK pathway in histiocytic sarcoma. Mod. Pathol..

[4] Egan C (2020). Genomic profiling of primary histiocytic sarcoma reveals two molecular subgroups. Haematologica.

[5] Dalia S, Jaglal M, Chervenick P, Cualing H, Sokol L (2014). Clinicopathologic characteristics and outcomes of histiocytic and dendritic cell neoplasms: The Moffitt Cancer Center experience over the last twenty five years. Cancers (Basel).

[6] Takada M (2018). Targeting MEK in a translational model of histiocytic sarcoma. Mol. Cancer Ther..

[7] Takada M (2019). Development of an orthotopic intrasplenic xenograft mouse model of canine histiocytic sarcoma and its use in evaluating the efficacy of treatment with dasatinib. Comp. Med..

[8] Thaiwong T, Sirivisoot S, Takada M, Yuzbasiyan-Gurkan V, Kiupel M (2018). Gain-of-function mutation in PTPN11 in histiocytic sarcomas of Bernese Mountain Dogs. Vet. Comp. Oncol..

[9] Takahashi M (2014). Clinical characteristics and prognostic factors in dogs with histiocytic sarcomas in Japan. J. Vet. Med. Sci..

[10] Craig LE, Julian ME, Ferracone JD (2002). The diagnosis and prognosis of synovial tumors in dogs: 35 cases. Vet. Pathol..

[11] Rassnick KM (2010). Phase II, open-label trial of single-agent CCNU in dogs with previously untreated histiocytic sarcoma. J. Vet. Intern. Med..

[12] Skorupski KA (2007). CCNU for the treatment of dogs with histiocytic sarcoma. J. Vet. Intern. Med..

[13] Asada H (2017). A 2-base insertion in exon 5 is a common mutation of the TP53 gene in dogs with histiocytic sarcoma. J. Vet. Med. Sci..

[14] Katoh M, Igarashi M, Fukuda H, Nakagama H, Katoh M (2013). Cancer genetics and genomics of human FOX family genes. Cancer Lett..

[15] Cortes JE (2012). Ponatinib in refractory Philadelphia chromosome-positive leukemias. N. Engl. J. Med..

[16] Michonneau D (2014). BRAF(V600E) mutation in a histiocytic sarcoma arising from hairy cell leukemia. J. Clin. Oncol..

[17] Kordes M (2016). Cooperation of BRAF(F595L) and mutant HRAS in histiocytic sarcoma provides new insights into oncogenic BRAF signaling. Leukemia.

[18] Liu Q (2016). Somatic mutations in histiocytic sarcoma identified by next generation sequencing. Virchows Arch..

[19] Dachy G (2019). Association of PDGFRB mutations with pediatric myofibroma and myofibromatosis. JAMA Dermatol..

[20] Roszik J (2019). Unique aberrations in intimal sarcoma identified by next-generation sequencing as potential therapy targets. Cancers (Basel).

[21] Vega F, Medeiros LJ, Bueso-Ramos CE, Arboleda P, Miranda RN (2015). Hematolymphoid neoplasms associated with rearrangements of PDGFRA, PDGFRB, and FGFR1. Am. J. Clin. Pathol..

[22] Tani H (2020). Canine histiocytic sarcoma cell lines with SHP2 p.Glu76Gln or p.Glu76Ala mutations are sensitive to allosteric SHP2 inhibitor SHP099. Vet. Comp. Oncol..

[23] Szymkiewicz I (2002). CIN85 participates in Cbl-b-mediated down-regulation of receptor tyrosine kinases. J. Biol. Chem..

[24] Hanahan D, Weinberg RA (2000). The hallmarks of cancer. Cell.

[25] Menyhárt O (2016). Guidelines for the selection of functional assays to evaluate the hallmarks of cancer. Biochim. Biophys. Acta Rev. Cancer.

[26] Cox KE, Maréchal A, Flynn RL (2016). SMARCAL1 resolves replication stress at ALT telomeres. Cell Rep..

[27] Hu G (2019). FOXM1 promotes hepatocellular carcinoma progression by regulating KIF4A expression. J. Exp. Clin. Cancer Res..

[28] Shukla S (2019). The FOXM1 inhibitor RCM-1 decreases carcinogenesis and nuclear β-catenin. Mol. Cancer Ther..

[29] Scott AJ, Lieu CH, Messersmith WA (2016). Therapeutic approaches to RAS mutation. Cancer J. (United States).

[30] Prior IA, Lewis PD, Mattos C, UKPMC Funders Group (2012). A comprehensive survey of Ras mutations in cancer. Cancer Res..

[31] Paik PK (2017). A phase Ib open-label multicenter study of AZD4547 in patients with advanced squamous cell lung cancers. Clin. Cancer Res..

[32] SenthilKumar G (2020). FGFR inhibition enhances sensitivity to radiation in non-small cell lung cancer. Mol. Cancer Ther..

[33] Lam W-S (2020). A phase II trial of single oral FGF inhibitor, AZD4547, as second or third line therapy in malignant pleural mesothelioma. Lung Cancer.

[34] Laurie SA (2017). A phase II trial of dovitinib in previously-treated advanced pleural mesothelioma: The Ontario Clinical Oncology Group. Lung Cancer.

[35] Schliemann C (2016). A phase I dose escalation study of the triple angiokinase inhibitor nintedanib combined with low-dose cytarabine in elderly patients with acute myeloid leukemia. PLoS One.

[36] Wu Q (2016). SCLLTargeting FGFR1 to suppress leukemogenesis in syndromic and de novo AML in murine models. Oncotarget.

[37] Procopio M-G (2015). Combined CSL and p53 downregulation promotes cancer-associated fibroblast activation. Nat. Cell Biol..

[38] Chen Z (2019). C11, a novel fibroblast growth factor receptor 1 (FGFR1) inhibitor, suppresses breast cancer metastasis and angiogenesis. Acta Pharmacol. Sin..

[39] Xiao L (2015). Endostar attenuates melanoma tumor growth via its interruption of b-FGF mediated angiogenesis. Cancer Lett..

[40] Holdman XB (2015). Upregulation of EGFR signaling is correlated with tumor stroma remodeling and tumor recurrence in FGFR1-driven breast cancer. Breast Cancer Res..

[41] Liu L (2014). Reductions in myeloid-derived suppressor cells and lung metastases using AZD4547 treatment of a metastatic murine breast tumor model. Cell. Physiol. Biochem..

[42] Ye T (2014). Inhibition of FGFR signaling by PD173074 improves antitumor immunity and impairs breast cancer metastasis. Breast Cancer Res. Treat..

[43] Katoh M (2016). FGFR inhibitors: Effects on cancer cells, tumor microenvironment and whole-body homeostasis (Review). Int. J. Mol. Med..

[44] Affolter VK, Moore PF (2002). Localized and disseminated histiocytic sarcoma of dendritic cell origin in dogs. Vet. Pathol..

[45] Tani A (2022). Changes in gene expression profiles and cytokine secretions in peripheral monocytes by treatment with small extracellular vesicles derived from a canine lymphoma cell line. J. Vet. Med. Sci..

[46] Koboldt DC (2012). VarScan 2: Somatic mutation and copy number alteration discovery in cancer by exome sequencing. Genome Res..

[47] Ravi N (2019). Identification of targetable lesions in anaplastic thyroid cancer by genome profiling. Cancers (Basel).

[48] Maeda S (2018). Comprehensive gene expression analysis of canine invasive urothelial bladder carcinoma by RNA-Seq. BMC Cancer.

[49] Asada H (2015). Evaluation of the drug sensitivity and expression of 16 drug resistance-related genes in canine histiocytic sarcoma cell lines. J. Vet. Med. Sci..

[50] Peters IR, Peeters D, Helps CR, Day MJ (2007). Development and application of multiple internal reference (housekeeper) gene assays for accurate normalisation of canine gene expression studies. Vet. Immunol. Immunopathol..

[51] Schweiger N (2015). Canine and human sarcomas exhibit predominant FGFR1 expression and impaired viability after inhibition of signaling. Mol. Carcinog..

[52] Takenaka H, Yasuno H, Kishimoto S (2002). Immunolocalization of fibroblast growth factor receptors in normal and wounded human skin. Arch. Dermatol. Res..

[53] Haq F (2018). FGFR1 expression defines clinically distinct subtypes in pancreatic cancer. J. Transl. Med..

[54] Azakami D (2006). Establishment and biological characterization of canine histiocytic sarcoma cell lines. J. Vet. Med. Sci..

[55] Ito K (2013). Identification of dasatinib as an in vitro potent growth inhibitor of canine histiocytic sarcoma cells. Vet. J..

