# Supplementary material for: Whole exome and transcriptome analysis revealed the activation of ERK and Akt signaling pathway in canine histiocytic sarcoma
Source: Sci Rep. 2023 May 25;13:8512. doi: 10.1038/s41598-023-35813-1 (PMC10212919; doi:10.1038/s41598-023-35813-1)
Supplement: Supplementary file 5 — Supplementary Information 5. [file 41598_2023_35813_MOESM5_ESM.pdf]

Table S1. Performance metrics in whole exome sequencing (WES) and RNA-seq.

Sample		WES					RNA-seq			
		Total reads (M)	Trimmed reads (M)	Mapped reads (M)	Mapping rate (%)	Coverage	RIN	Total reads (M)	Mapped reads (M)	Mapping rate (%)
Dog 1	N	193.5	185.4	184.2	99.3	427.6	9.3	62.8	60.0	95.6
	T	178.1	170.0	167.7	99.6	396.2				
Dog 2	N	199.0	190.9	189.8	99.4	440.1	8.2	37.7	31.1	82.5
	T	175.3	168.2	167.0	99.3	387.9				
Dog 3	N	199.2	191.0	184.9	99.2	440.7	9.6	43.5	36.2	83.4
	T	195.1	186.6	184.9	99.1	431.6				
Dog 4	N	119.8	92.0	91.4	99.4	318.1	8.7	42.5	34.9	82.1
	T	111.5	85.3	84.7	99.3	295.1				
Dog 5	N	133.3	98.7	97.9	99.2	341.5	NE	NE	NE	NE
	T	65.8	76.0	75.2	99.0	262.7				
PBMoC 1		NE	NE	NE	NE	NE	8.8	102.7	92.7	90.3
PBMoC 2		NE	NE	NE	NE	NE	8.0	103.5	92.5	89.4
PBMoC 3		NE	NE	NE	NE	NE	9.0	109.6	98.2	89.6

N, normal cell sample; T, tumor cell sample; RIN, RNA integrity number; NE, not evaluated

Table S2. Normalized expression values of receptor tyrosine kinase genes in data of RNA-seq.

Dog 1		Dog 2		Dog 3		Dog 4	
Gene	Normalized expression value	Gene	Normalized expression value	Gene	Normalized expression value	Gene	Normalized expression value
FGFR1	121.6	*PDGFRB*	137.4	*PDGFRB*	59.0	*FGFR1*	18.7
VEGFR1	39.5	*FGFR1*	128.5	*FGFR1*	18.5	*FLT3*	2.2
PDGFRA	25.6	*ERBB2*	59.9	*VEGFR2*	16.7	*PDGFRB*	1.5
ERBB2	24.1	*NTRK3*	31.0	*VEGFR3*	15.4	*ERBB2*	1.2
ERBB1	17.1	*VEGFR3*	30.1	*ERBB2*	10.4	*VEGFR3*	1.0
PDGFRB	6.0	*VEGFR2*	25.5	*ERBB1*	8.8	*ERBB3*	0.9
FGFR3	2.3	*ERBB1*	22.8	*VEGFR1*	6.7	*FGFR3*	0.8
FLT3	1.0	*VEGFR1*	16.4	*ERBB3*	6.0	*PDGFRA*	0.3
FGFR2	0.7	*FGFR3*	15.7	*PDGFRA*	5.6	*NTRK3*	0.0
VEGFR3	0.4	*PDGFRA*	13.5	*FLT3*	4.8	*RET*	0.0
ERBB3	0.4	*FGFR2*	9.0	*NTRK3*	2.4	*FGFR4*	0.0
NTRK1	0.2	*RET*	1.6	*FGFR3*	2.4	*VEGFR2*	0.0
NTRK3	0.1	*FLT3*	1.3	*RET*	2.1	*ERBB1*	0.0
RET	0.1	*ERBB3*	1.2	*KIT*	1.7	*VEGFR1*	0.0
FGFR4	0.1	*KIT*	1.1	*NTRK1*	1.3	*KIT*	0.0
VEGFR2	0.0	*FGFR4*	0.9	*NTRK2*	1.3	*NTRK1*	0.0
KIT	0.0	*NTRK1*	0.8	*FGFR2*	0.2	*NTRK2*	0.0
NTRK2	0.0	*NTRK2*	0.0	*FGFR4*	0.0	*FGFR2*	0.0

Table S3. Information of canine histiocytic sarcoma cases included in this study.

Dog	Breed	Sex	Age (y)	Subtype	Lesion locations	Obtained tumor cell samples	Sample type	Normal cell samples	WES	RNA-seq	RT-qPCR	IHC	Cell line
1	Shiba	SF	12	DHS	Soft tissue and mandibular LN	Soft tissue mass	Frozen/FFPE	PB	○	○	○	○	DHS-1
2	Pomeranian	M	12	DHS	Spleen and liver	Liver mass	Frozen/FFPE	Normal region of the liver	○	○	○	○	-
3	Flat-coated Retriever	M	8	DHS	Shoulder joint and skin	Shoulder joint mass	Frozen/FFPE	Normal skin	○	○	○	○	-
4	Miniature Schnauzer	F	6	LHS	Lung	Pleural fluid	Frozen	PB	○	○	○	-	DHS-2
5	Flat-coated Retriever	F	11	LHS	Lung	Lung mass	Frozen/FFPE	PB	○	-	-	○	-
6	Flat-coated Retriever	CM	8	DHS	Soft tissue, LN	Soft tissue mass	Frozen	-	-	-	○	-	-
7	Bernese Mountain Dog	CM	8	DHS	Lung (multiple lesions)	Lung mass	Frozen	-	-	-	○	-	-
8	Golden Retriever	CM	13	LHS	Elbow joint	Elbow joint mass	Frozen	-	-	-	○	-	-
9	Beagle	CM	9	DHS	Spleen and soft tissue	Spleen	FFPE	-	-	-	-	○	-
10	Flat-coated Retriever	SF	6	DHS	Lung, LN	Lung mass	FFPE	-	-	-	-	○	-
11	Welsh Corgi Pembroke	CM	8	DHS	Lung (multiple lesions)	Lung mass	FFPE	-	-	-	-	○	-
12	Rottweiler	CM	9	DHS	Systemic organs	Urinary bladder mass	FFPE	-	-	-	-	○	-
13	Flat-coated Retriever	CM	9	DHS	Liver, spleen, and LN	LN	FFPE	-	-	-	-	○	-
14	Welsh Corgi Pembroke	M	11	LHS	Lung	Lung mass	FFPE	-	-	-	-	○	-
15	Labrador Retriever	SF	15	LHS	Soft tissue	Soft tissue mass	FFPE	-	-	-	-	○	-
16	Pointer	SF	12	DHS	Spleen and lung	Spleen	FFPE	-	-	-	-	○	-
17	Welsh Corgi Pembroke	CM	12	DHS	Lung (multiple lesions)	Lung mass	FFPE	-	-	-	-	○	-
18	Beagle	CM	6	-	-	-	PB	PB	-	○	○	-	-
19	Beagle	CM	7	-	-	-	PB	PB	-	-	○	-	-
20	Beagle	CM	7	-	-	-	PB	PB	-	-	○	-	-

M, male; CM, castrated male; F, female; SF, spayed female; DHS, disseminated histiocytic sarcoma; LHS, localized histiocytic sarcoma;

LN, lymph node; FFPE, formalin-fixed paraffin-embedded; PB, peripheral blood; WES, whole exome sequencing; RNA-seq, RNA-sequencing; IHC, immunohistochemistry

Table S4. IC_{50} values of cell lines against ponatinib.

	CHS1	CHS2	CHS3	CHS4	CHS5	CHS6	CHS7	CHS8	MHT2	DH82	DHS1	DHS2	MDCK
IC_{50} (nM)	1090	490	454	321	659	132	462	944	779	1350	2200	90.4	-

Table S5. Antibodies used in this study.

Target	Manufacturer	Host and clonality	clone	Catalog no.	RRID	Dilution	
						WB	IHC
CD14	Abcam	Rabbit mAb	Tuk4	ab27545	AB_2857907	-	-
FGFR1	Abcam	Rabbit pAb	-	ab10646	AB_297367	-	1:1,500
ERK1/2	CST	Rabbit mAb	137F5	4695	AB_390779	1:1,000	-
pERK1/2 (Y202/Y204)	CST	Rabbit mAb	D13.14.4E	4370	AB_2315112	1:2,000	-
Akt	CST	Rabbit mAb	C67E7	4691	AB_915783	1:1,000	-
pAkt (S473)	CST	Rabbit mAb	D9E	4060	AB_2315049	1:2,000	-
β-actin	Novus Biologicals	Mouse mAb	ac-15	NB600-501	AB_10077656	1:10000	-
Mouse IgG	Tokyo Chemical Industry	Goat pAb-HRP	-	G0407	AB_2857910	1:3000	-

CST, Cell Signalling Technology; mAb, monoclonal antibody; pAb, polyclonal antibody; HRP, horseradish peroxidase

Table S6. Research resource identifier for statistical tool and software used in this study.

Name	RRID
bcl2fastq	SCR_015058
Trimmomatic	SCR_011848
Bowtie 2	SCR_005476
Genotype Analysis Toolkit	SCR_001876
SnpEff	SCR_005191
Polyphen-2	SCR_013189
SIFT	SCR_012813
PROVEAN	SCR_002182
DAVID Bioinformatics Resource	SCR_001881
EdgeR	SCR_012802
Java TreeView	SCR_016916
Ingenuity Pathway Analysis	SCR_008653
Primer3	SCR_003139
ImageJ	SCR_003070
Prism	SCR_002798

Table S7. Primers used for validation of the results of whole exome sequeneces by Sanger sequencing.

Dog	Gene	Forward primer (5′-)	Reverse primer (5′-)
Dog 1	*CCDC136*	CACTGTGAGGACATGGTTGC	AGGCAGTGGGCTTCTCAAG
	TP53	ACCCCCACCCAATACCTG	GCCTTGTCCCATCTGTAG
Dog 2	*BBX*	AGAAGGAGAAAGGGCTGGAA	GGATGCAAGTTATGCCCACT
	N4BP2	GGAAACTCAGAGCAGGCAGA	TCTTCAGTGCTTGGAAATCTCA
	PDGFRB	AGCCAGAAACGTGCTCATCT	TCTCCCATGTGTCACAGTCA
Dog 3	*ATRX*	TCTTCCTGCGCATGTAAATCA	TGCATGTATGTTTGGCACTCC
	GARNL3	GTCTCGGCCCTAGGATTTTC	GACTGGGGTAAGGTCCTGTG
	HTR2C	GATATTTGTGCCCCGTCTGG	TGTTACCAGTCGACGTCTGT
	NR3C2	GTGCAGGGAAGCTCATGTC	TTTCAGGGTCTCCTGCAACT
	NRXN3	TGCTCAACTACGGCTACGTG	ACCCCTCTCACAGGTCCTTC
	SH3KBP1	CAGAGAGAGAGCCAAAACTGG	CCGGAGTCTTCAGACAGCTT
	TP53	GCCCTGGTATAATGTTGCTGG	TCAGTGCTGGTTTTGCTTCC
Dog 4	*NAV3*	AAAGGGCCTCAACCATCTTC	GCGTTGACCAGCTTGAATTT
	PTPN11	GGCAGTGTAGCCCTTGAAAC	CCTGCTCTTCCTCAATCCTG
	ZMYM3	CTGTTTCAGCTGAGCCATCC	AATGCGCACAGGTCATCTTG
Dog 5	*AKAP4*	CCAGGACAAACAAAGCAGCT	CTTGACTACACCTGGAGCCA
	ASMT	ATGTGTTTGTGTTGCAGCCA	GAGGTCACCAGCCCTGAG
	C7	GCCCCAGTCATGAAGGAGAT	GGTGTAATTTCGGCCCTGAC
	CLCA4	CCACCCTACCCTTCACCTCT	GGTTGTTTCTTTCCTTGCTGC
	COL11A1	CCCTGGTTAGGCAGCTTTTA	GGCTACTTGGCTTGACATCC
	FBN1	AGCCTGAGATTCGAGTCACT	CCACTGGCTTCTTCTTGGTG
	FRAS1	GCCAACATTTCCATATGCCCA	CCAGGGAAGAGTCAAGAAGAGA
	FREM2	CCAATTTCCTACTCGCGGTC	CGAATGCCCTCTTGTCCAAC
	GGNBP2	GGGACTGGGATGTGCAAATC	GTCTGCTTCTCCTCCCTCTG
	LAMA1	AACTGCTTGAGCAGCCAGAC	GTGTGTCCGTGGAAGTTGTG
	MLEC	TTCTGGGCGTGTTACTAGCA	GCATTGCCTTCTGTCACCAA
	MTX2	TTTGCTCTAGATCACTCATGCT	AACCACTTCGCGTCAGAAAA
	NUGGC	GAGCCAATTCCAGCACCTTG	TGCAGGGCCAAATACTACGA
	OPCML	TGCACAGTGTTAATGGCCAC	TCCATGTCACAGTTGGCTCT
	PDGFD	TTGAAGGGCAGATGAGATCC	TGGTTCCACAGCCACAGTTA
	PTPN11	TGGCTCCTTGGGTATGTTTC	CCTATGGCATGGAAGAGGTG
	RELN	GGGGAGCAAGTCTGTTCTGA	TTGCAACTCGCATCAATCTC
	RIPK1	GGGCTTCACTGAACCTCAGA	GAGGAGAATACCCCATGCTG
	SLC6A13	TCACAAGTGGATGCCCTTCT	CCCAAGGCCTGAATCCATTG
	SMARCAL1	GAGCAAAGCCATGGGAAGAG	ATCTCAAGCCCCAGACTCAC

Table S8. Primers used in qPCR in this study.

Gene	Forward primer (5′-)	Reverse primer (5′-)	Amplicon size (bp)	PCR efficiency (%)	R^2
ERBB1	ACTGACCTCCATGCTTTCGA	GTTATGTTCAGGCCGACGAC	95	97.8	1.000
ERBB2	CCTGCCTTCACTTCAACCAC	GCATGGATTCGAAGGTGTCC	87	106.9	0.999
ERBB3	CTAGGCCCAGTTCTCTCGAG	AGTCCTCATCTGGGGTTGTG	144	98	0.990
ERBB4	CAGCACGATTCCAGAAGCTC	CTTGCGTAGGGTGCCATTAC	85	101.3	0.994
FGFR1	TTGACCGGATCTACACCCAC	GCTTGTCCATTCGATGACCC	147	95.7	1.000
FGFR2	CCACAACCAAGAAGCCAGAC	ACTGTTACCTGTCTCCGCAA	85	102.6	0.997
FGFR3	ATCAGTGAGAGTGTGGAGGC	TCTCAGCCACGCCTATGAAA	109	103.8	0.996
FGFR4	CTGACCTTCGGACCCTACTC	AGGAGGTCATGGCAGAAGAC	86	105.3	0.995
FLT3	TCACAGGACATGGACGGAAA	GAGCCCTGAGATGTGATCCA	136	109.2	0.994
KIT	TGGGAAAACTTTGGGTGCTG	AGGGCTTCTCGTTCGGTTAA	138	106.9	0.997
NTRK1	GACGGAGCTCTACATCGACA	AGCCGAGGAGTGAAATGGAA	147	104.3	0.998
NTRK2	CTTTGTGCACCGAGATCTGG	CCACCGACCCTGTAGTAGTC	120	93.9	0.998
NTRK3	GATCACCTCTGCCCGATGTA	GATGGCATGTACGTTGGTCC	95	95.6	0.999
PDGFRA	TTCACCTATCAAGTTGCGCG	CACGATTTTCCCTTGTGCCA	105	101.8	1.000
PDGFRB	ACTGTGTCCACCGAGATCTG	CATCCACTTCAGAGGCAGGA	149	94.4	0.999
RET	CAGTGTTGGAAGCAGGAACC	GCCATCGTCATAAAGCAGGG	141	104.7	0.995
VEGFR1	AATGGACGAGGACTTCTGCA	GCCAGCAGTCCAACATGATC	101	98.1	0.999
VEGFR2	GTGCTTCTCCGTATCCTGGA	TGGGTCTCTGATTGGGTTCC	150	100.7	0.999
VEGFR3	TGTGTGTGAGGCCAACAATG	CCATTCGACGCTGATGAAGG	88	99.5	0.999
RPL32	TGGTTACAGGAGCAACAAGAAA	GCACATCAGCAGCACTTCA	100	95.7	0.990

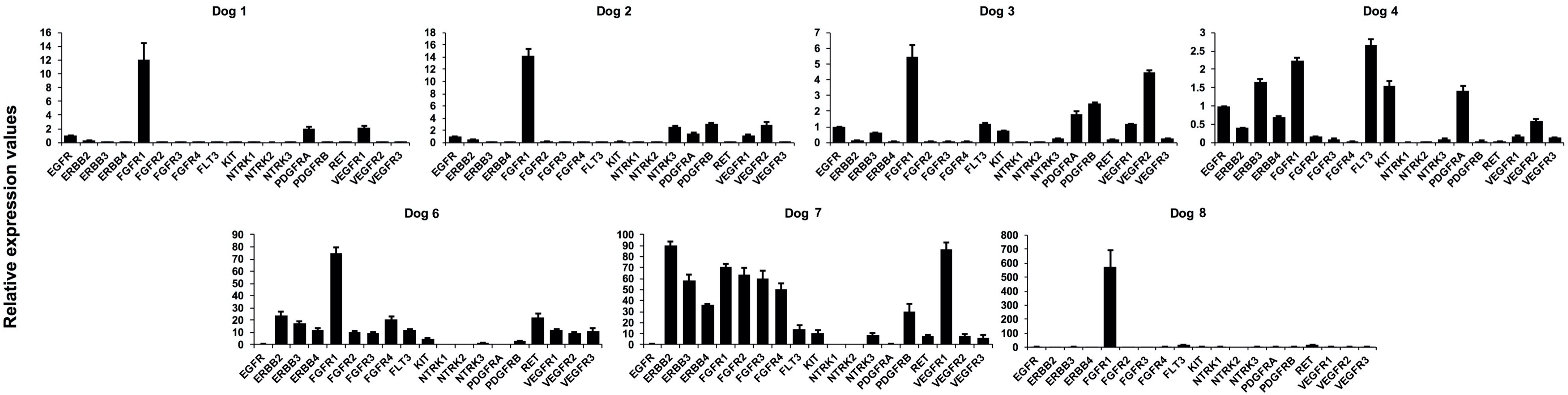

Fig. S1: Comparison of mRNA expressions among receptor tyrosine kinase (RTK) genes in seven canine histiocytic sarcoma cases. *FGFR1* mRNA expression was the highest among RTK genes in five of the seven cases. *RPL32* was used as an internal control gene. Each experiment was conducted in triplicate.

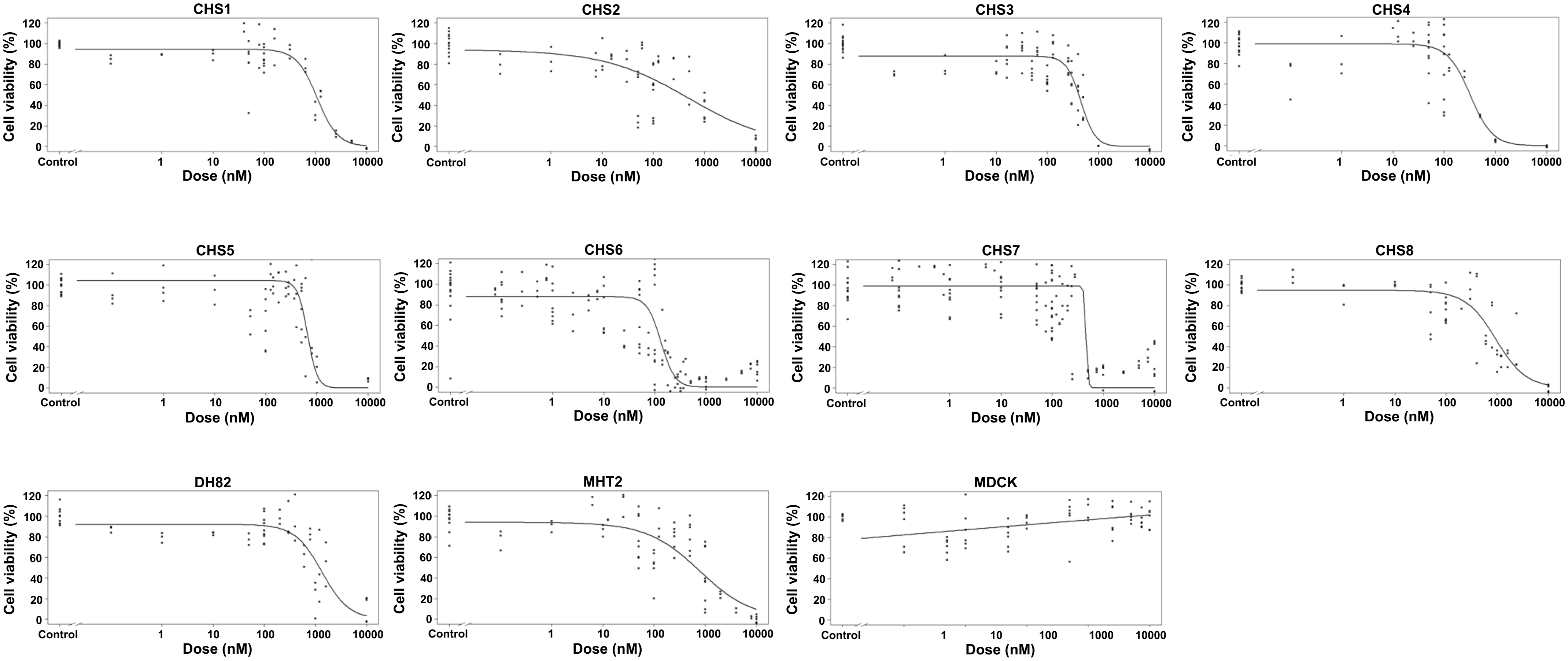

Fig. S2: Cell viability assays against ponatinib using 10 canine histiocytic sarcoma cell lines, CHS1, CHS2, CHS3, CHS4, CHS5, CHS6, CHS7, CHS8, DH82, MHT2, and non-HS cell, MDCK.

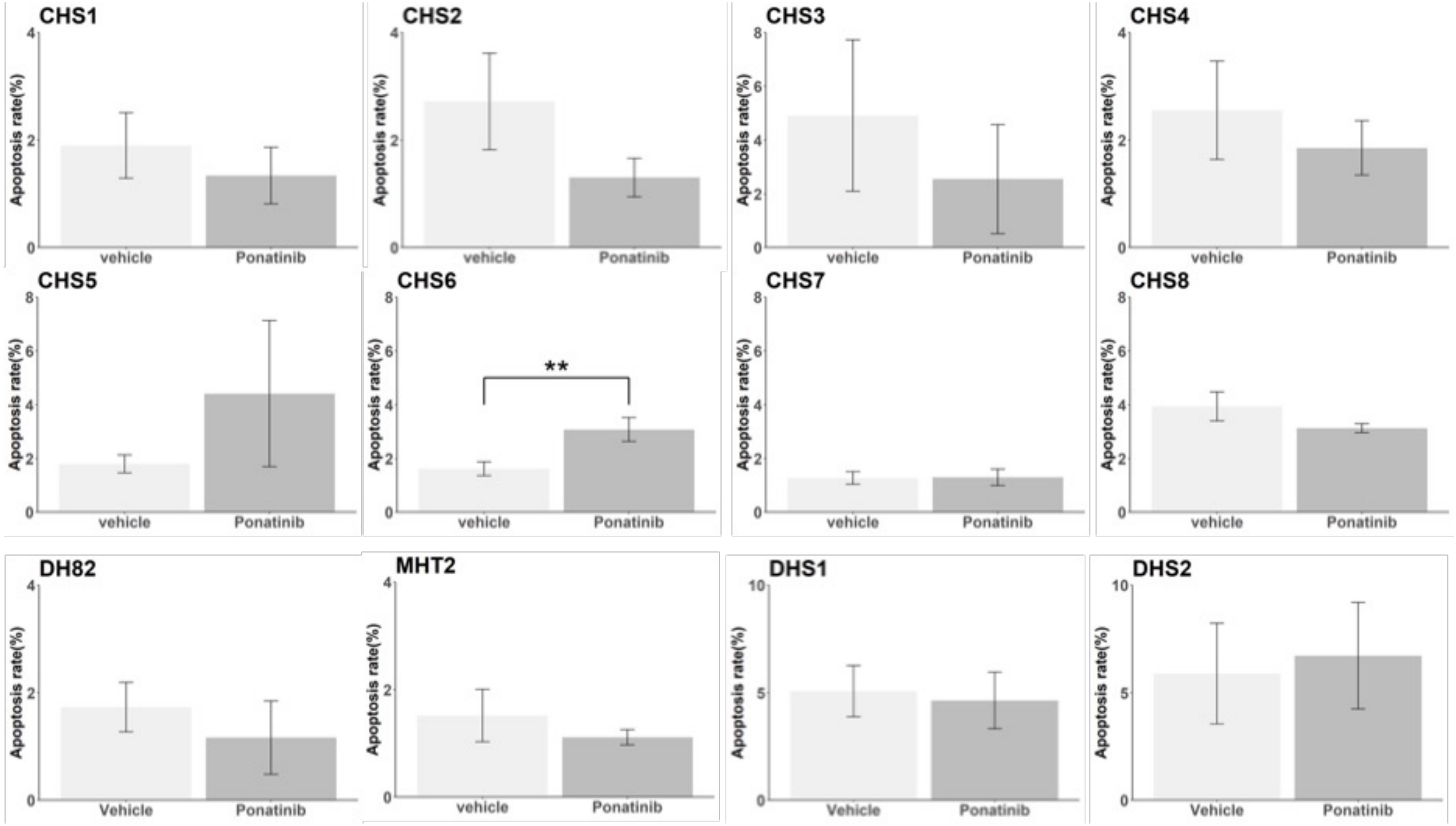

Fig. S3: Comparisons of the rates of cells showing apoptosis using unpaired t test in 12 canine histiocytic sarcoma cell lines, CHS1, CHS2, CHS3, CHS4, CHS5, CHS6, CHS7, CHS8, DH82, MHT2, DHS1, and DHS2. $^{**}P < 0.01$.

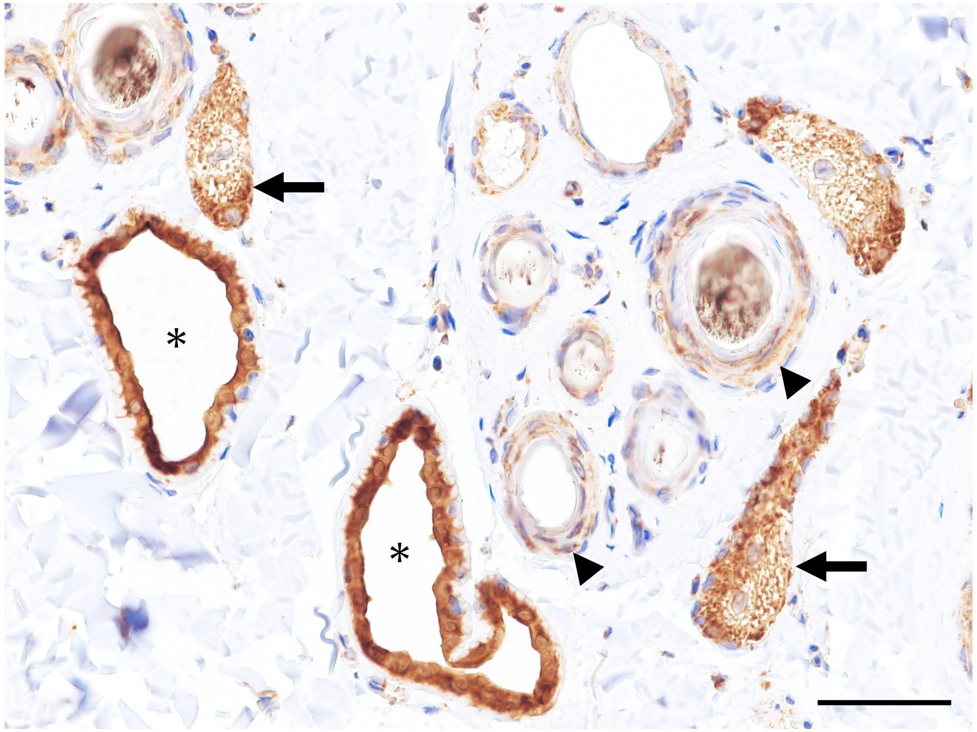

Fig. S4: Representative results of positive control for immunohistochemistory against FGFR1 using normal skin tissue of Dog 3. Epithelial cells of the hair follicles (arrowheads), sweat glands (*), and sebaceous glands (arrows) are positively immunolabeled with anti-FGFR1 antibody as expected. Bar, 50μm.

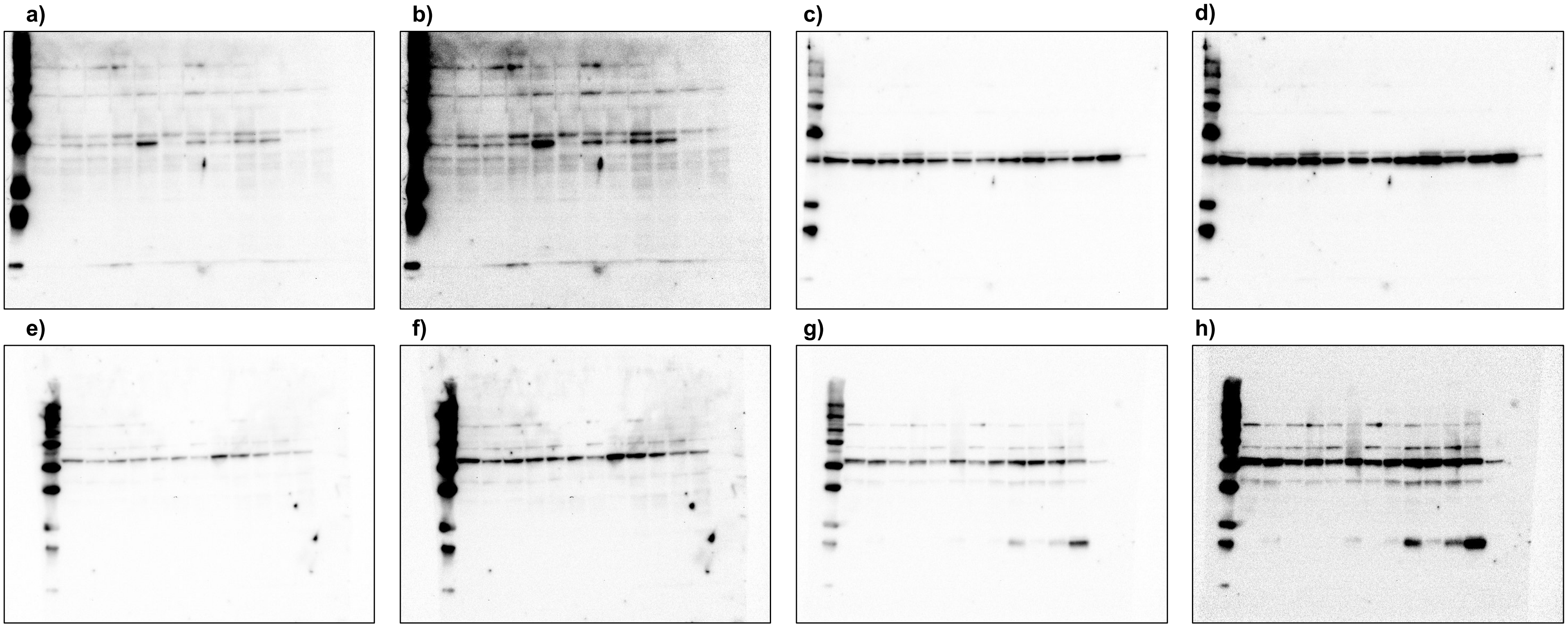

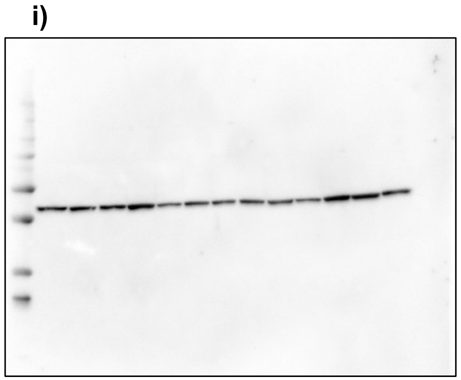

Fig. S5: Figures of full-length membrane that were used for Fig. 4a. These figures showed the blots for a) low exposure phosphorylated ERK1/2, b) high exposure phosphorylated ERK1/2, c) low exposure ERK1/2, d) high exposure ERK1/2, e) low exposure phosphorylated AKT, f) high exposure phosphorylated AKT, g) low exposure AKT, h) high exposure AKT, i) β-actin. The results of cell lysates derived from CHS1, CHS2, CHS3, CHS4, CHS5, CHS6, CHS7, CHS8, MHT2, DH82, DHS1, DHS2, peripheral blood CD14+ monocytes were shown following a marker lane.

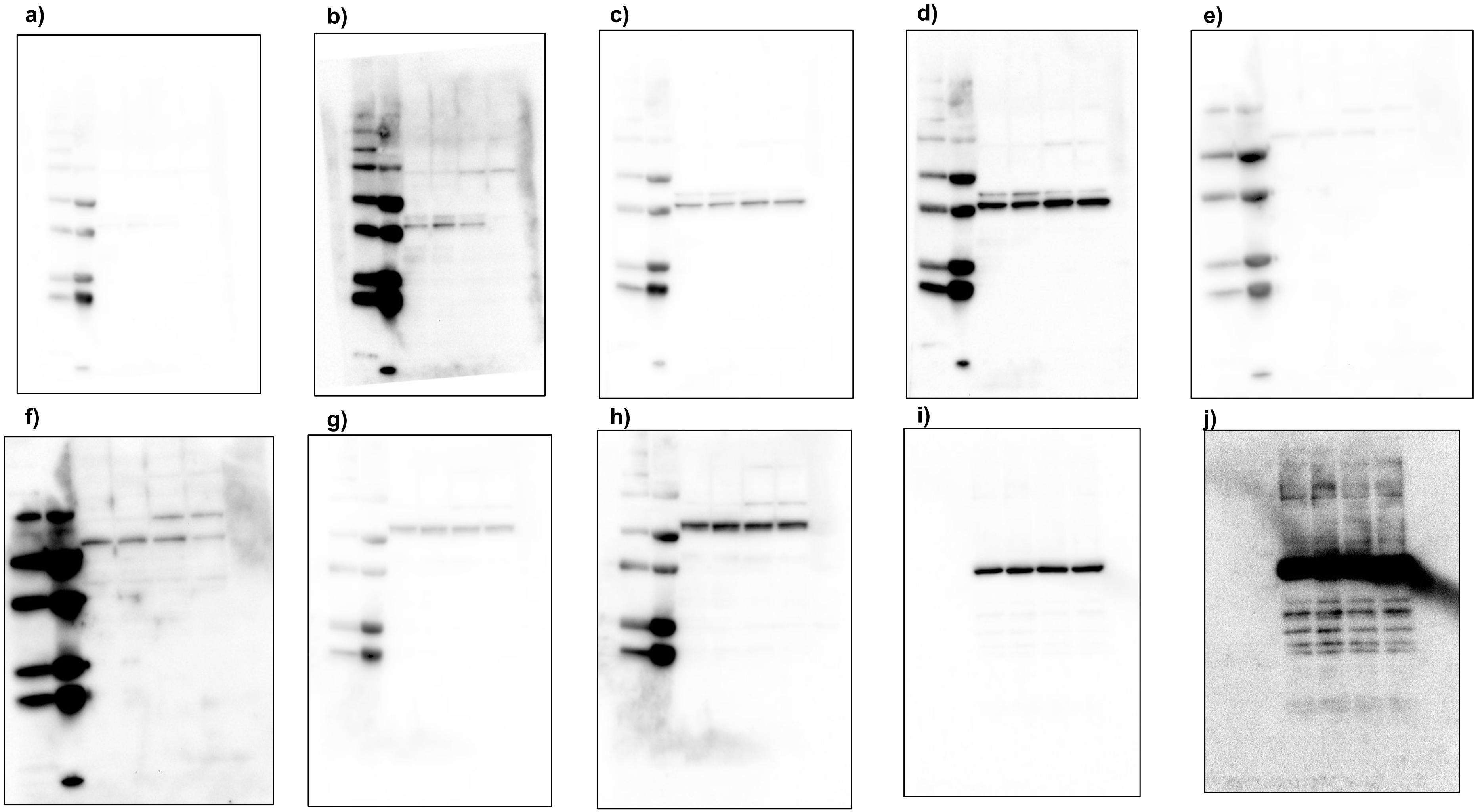

Fig. S6: Figures of full-length membrane that were used for Fig. 5b. These figures showed the blots for a) low exposure phosphorylated ERK1/2, b) high exposure phosphorylated ERK1/2, c) low exposure ERK, d) high exposure ERK, e) low exposure phosphorylated Akt, f) high exposure phosphorylated Akt, g) low exposure Akt, h) high exposure Akt, i) low exposure β-actin, and j) high exposure β-actin. In a)-h), the results of cell lysates derived from not-treated DHS1, ponatinib-treated DHS1, no-treated DHS2, ponatinib-treated DHS2 were shown following two marker lanes. Marker lanes were not included for β-actin I, j).
